# Validation and Reliability of the Thai Pediatric Charcot–Marie–Tooth Quality of Life Outcome Measure

**DOI:** 10.1111/jns.70075

**Published:** 2025-11-16

**Authors:** Pimchanok Kulsirichawaroj, Arisa Phochaisarn, Sivaporn Limpaninlachat, Nutchavadee Vorasan, Surachai Likasitwattanakul, Sindhu Ramchandren, Michael E. Shy, Oranee Sanmaneechai

**Affiliations:** ^1^ Division of Neurology, Department of Pediatrics, Faculty of Medicine Siriraj Hospital Mahidol University Bangkok Thailand; ^2^ Center of Research Excellence for Neuromuscular Diseases, Faculty of Medicine Siriraj Hospital Mahidol University Bangkok Thailand; ^3^ Faculty of Physical Therapy Mahidol University Bangkok Thailand; ^4^ Genetic Epidemiology Research Cluster, Research Division, Faculty of Medicine Siriraj Hospital Mahidol University Bangkok Thailand; ^5^ Department of Neurology Wayne State University Detroit Michigan USA; ^6^ Department of Neurology University of Iowa Hospitals and Clinics Iowa Iowa USA

**Keywords:** Charcot–Marie–tooth disease, patient‐reported outcome measures, quality of life, Thai pCMT‐QoL, translation

## Abstract

**Background and Aims:**

Charcot–Marie–Tooth disease (CMT) is a hereditary neuropathy that causes progressive muscle weakness, sensory deficits, and impaired mobility, significantly affecting quality of life (QoL). The Pediatric Charcot–Marie–Tooth Quality of Life (pCMT‐QoL) instrument was developed specifically for children with CMT. However, a validated Thai version is not yet available.

**Methods:**

We conducted a cross‐sectional study at the Pediatric Neuromuscular Clinic from July 2023 to December 2024. Using a forward–backward translation method, we adapted the pCMT‐QoL into Thai. Twenty‐three children with CMT and their caregivers completed the Thai questionnaire. We evaluated internal consistency using Cronbach's alpha and test–retest reliability using intraclass correlation coefficients (ICCs). Convergent validity was examined via Pearson correlation between child self‐reports and parent‐proxy reports across functional, mental, and physical domains.

**Results:**

The Thai pCMT‐QoL demonstrated high test–retest reliability (ICC > 0.85) and satisfactory internal consistency (Cronbach's alpha > 0.7) across most domains. Convergent validity was strong for the total and mental domains but weaker for the physical domain, reflecting differences in perception between children and parents. Parents generally reported higher QoL scores than children did, a finding consistent with studies in other neuromuscular diseases. Most participants completed the questionnaire within 15 min, suggesting good feasibility.

**Interpretation:**

The Thai pCMT‐QoL is a reliable, culturally adapted tool for assessing QoL in children with CMT. It is suitable for both remote and in‐clinic administration. Future studies with larger cohorts are needed to confirm its responsiveness to clinical changes and to broaden its application in diverse settings.

## 1 Introduction

Charcot–Marie–Tooth disease (CMT), also known as hereditary motor and sensory polyneuropathy, is a hereditary peripheral neuropathy that affects about 1 in 2500 individuals [1]. It is characterized by progressive muscle weakness, sensory loss, and foot deformities. More than 100 implicated genes confer substantial genetic and phenotypic heterogeneity [2]. This complexity can profoundly affect patients' quality of life (QoL).

Recent investigations into CMT therapies highlight the importance of early‐stage interventions, especially in pediatric populations. To support clinical trial readiness, researchers have developed CMT‐specific outcome measures and categorized them into clinical outcomes, functional outcomes, patient‐reported outcomes, biomarkers of disease burden, and treatment‐specific biomarkers of target engagement [3].

Clinical and functional outcome measures for pediatric CMT, such as the CMT Pediatric Scale and the CMT Infant Scale, are available worldwide [4, 5]. However, patient‐reported and self‐reported outcome measures remain limited. The Pediatric Charcot–Marie–Tooth Quality of Life (pCMT‐QoL) instrument was developed as a disease‐specific tool, with versions in English and Italian [6, 7, 8, 9]. The original English version of the pCMT‐QoL comprises three instruments [6, 8, 10]. These instruments include a parent‐report version for children aged 0–7 years, a parent‐report version for children aged 8–18 years, and a child self‐report version for children aged 8–18 years.

In Thailand, several studies have described the clinical spectrum, gene distribution, and genotype–phenotype correlation of pediatric CMT [11, 12]. However, no validated Thai QoL assessment tool exists specifically for this population. This deficiency hinders accurate evaluation of disease burden and the effectiveness of therapeutic interventions.

This study aimed to establish the validity and reliability of a Thai version of the pCMT‐QoL as a clinically useful measurement for Thai physicians. Our findings should enhance understanding of QoL in pediatric CMT and provide valuable patient‐reported data for international drug trials targeting this rare disease.

## 2 Materials and Methods

### 2.1 Study Design and Patient Population

This cross‐sectional study was conducted at the Pediatric Neuromuscular Clinic at Siriraj Hospital, Thailand, from July 2023 to December 2024. We included children aged 0 to 18 years with a confirmed diagnosis of CMT by electrophysiological testing or nerve conduction studies. All patients underwent genetic testing. The Siriraj Institutional Review Board approved the study protocol (354/2566 [IRB3] and COA no. Si 446/2023). The research was conducted in accordance with the principles of the Declaration of Helsinki. Written informed consent was obtained from all participating children and their parents or caregivers.

Each patient and parent or caregiver independently completed the Thai pCMT‐QoL (Supporting Information [Link], [Link], [Link]). For participants with physical or visual limitations, a research assistant read the questions aloud and recorded the responses. The working version of the pCMT‐QoL was administered twice, separated by a 2–4‐week interval. The first administration took place in the clinic, and the second was mailed to participants' homes.

We collected patients' demographic information, education level, ambulatory status, surgical history, electrophysiologic subtype, and genetic findings. For parents, we recorded demographic data, education level, marital status, socioeconomic status, employment status, and household income.

### 2.2 Measures and Procedures

We obtained formal permission from the original developers (S.R. and M.S.) to use and translate the pCMT‐QoL instrument. The translation process followed established linguistic guidelines and used a forward–backward method [13, 14].

Two certified Thai translators, both fluent in English, performed the initial forward translation. Three pediatric neurologists with extensive experience in CMT then reviewed and refined this draft version.

Next, two additional certified translators, who were blinded to the original version, back‐translated the revised text into English (Appendices A, B, C). We compared the back‐translated copy with the original pCMT‐QoL to confirm conceptual accuracy and resolve any inconsistencies. The finalized Thai version of the pCMT‐QoL (Supporting Information [Link], [Link], [Link]) was then implemented in the study.

In addition to the pCMT‐QoL, we administered the Thai version of the Pediatric Quality of Life Inventory (PedsQL 4.0) Generic Core Scales on the same day that patients and caregivers completed the first pCMT‐QoL assessment. The 23‐item PedsQL 4.0 is a well‐established, validated instrument designed to measure health‐related quality of life in children and adolescents [15, 16]. It includes four multidimensional scales: Physical Functioning (8 items), Emotional Functioning (5 items), Social Functioning (5 items), and School Functioning (5 items). Items are rated on a 5‐point Likert scale (0 = never a problem to 4 = almost always a problem), with scores transformed on a scale from 0 to 100, where higher scores indicate better functioning. The tool is available in both child self‐report and parent‐proxy versions and was used to evaluate convergent validity by correlating domain scores with those of the Thai pCMT‐QoL.

### 2.3 pCMT‐QoL Instrument

The pCMT‐QoL instrument contains 57 items across six domains: symptoms (12 items), function (10), social activities (7), feelings (10), cognition (10), and social skills (8). The first three domains reflect physical aspects, and the last three address mental aspects.

Child self‐reports and parent‐proxy versions are available for children aged 8–18 years, while only parent‐proxy versions exist for children aged 0–7 years. Both versions feature identical core items, adjusted for age‐appropriate language and first‐ or third‐person tense. Each item is scored on a 5‐point scale (0 = never a problem, 1 = almost never a problem, 2 = sometimes a problem, 3 = almost always a problem, and 4 = always a problem), with reverse scoring for positively framed items.

Scoring proceeds in two steps. First, a weighted sum of the domain items is calculated based on dataset‐derived weights. Then, the summed scores are converted to a 0‐to‐100 scale, where 100 indicates the worst QoL and 0 indicates the best. Study data were collected and managed through REDCap (Research Electronic Data Capture), a secure, web‐based application designed to support validated data entry, audit trails, streamlined data export, and data import from external sources [17]. The system was hosted at the Research Data Management Unit, Faculty of Medicine Siriraj Hospital, Mahidol University.

### 2.4 Statistical Analysis

All analyses were performed using IBM SPSS Statistics, version 29 (IBM Corp., Armonk, NY, USA). Statistical significance was set at *p* < 0.05. Demographic data are reported as frequencies, percentages, means, and standard deviations. We used a *t* test to assess mean differences between groups.

#### 2.4.1 Test–Retest Reliability

Parent–child dyads completed the pCMT‐QoL instrument on two separate occasions, spaced 2–4 weeks apart. We evaluated test–retest reliability using intraclass correlation coefficients (ICCs). According to established guidelines, ICC < 0.5 indicates poor reliability, 0.5–0.75 indicates moderate reliability, 0.75–0.9 indicates good reliability, and > 0.90 indicates excellent reliability [18, 19].

#### 2.4.2 Internal Consistency

We assessed the internal consistency of each pCMT‐QoL domain using Cronbach's alpha. Values above 0.7 were considered acceptable, reflecting satisfactory internal consistency [20].

#### 2.4.3 Convergent Validity

We examined convergent validity using Pearson correlations in two ways. First, we assessed the correlations between parent‐proxy and child self‐reported pCMT‐QoL scores (ages 8–18), including total scores, physical composite domain scores, and mental composite domain scores. Second, to further support convergent validity using an independent measure, we assessed correlations between corresponding domain scores of the Thai pCMT‐QoL and the Thai version of PedsQL 4.0 Generic Core Scales (Physical, Emotional, Social, and School Functioning). Correlations were classified as negligible (0.00–0.10), weak (0.10–0.39), moderate (0.40–0.69), strong (0.70–0.89), or very strong (0.90–1.00) [21].

## 3 Results

### 3.1 Demographic Characteristics

We enrolled 23 pediatric patients with CMT and their parents. Among these participants, five had children aged 0–7 years, and 18 had children aged 8–18 years, corresponding to the respective versions of the Thai CMT‐QoL. The mean age of the children was 12.1 years, with an average symptom onset at 4.6 years. Most children (60.9%) had the axonal subtype of CMT. A majority of caregivers were married, had completed secondary school or held a bachelor's degree, and were employed (87.0%). Nearly half reported a household income of less than 10 000 baht per month (Table 1 and Table 2).

**TABLE 1:** Demographic characteristics of pediatric patients with Charcot–Marie–Tooth disease.

Characteristics	*N* = 23
Sex, male	13 (56.5%)
Age at evaluation (years)	12.1 ± 4.7
Age at onset (years)	4.6 ± 4.9
Previous foot surgery	4 (17.4%)
Non‐ambulatory patients	3 (13.0%)
Education level	
Kindergarten	4 (17.4%)
Primary school	7 (30.4%)
Secondary school	11 (47.8%)
Bachelor's degree	1 (4.3%)
Family history	4 (17.4%)
Electrophysiologic subtype	
Axonal	14 (60.9%)
Demyelinating	7 (30.4%)
Intermediate	1 (4.3%)
Undetermined	1 (4.3%)

*Note:* Data are displayed as either mean ± SD or *n* (%).

**TABLE 2:** Demographic characteristics of parents and caregivers of pediatric patients with Charcot–Marie–Tooth disease.

Characteristics	*N* = 23
Sex, male	4 (17.4%)
Age (years)	47.5 ± 9.0
Marital status	
Single	3 (13.0)
Married	16 (69.6)
Divorced	4 (17.4)
Education level	
Primary school	3 (13.0)
Secondary school	8 (34.8)
Bachelor's degree	10 (43.5)
Master's degree	1 (4.3)
Doctoral degree	1 (4.3)
Employment status	
Employed	20 (87.0)
Unemployed	3 (13.0)
Household income per month in Thai baht	
< 10 000	10 (43.5)
10 000–30 000	9 (39.1)
30 000–50 000	3 (13.0)
> 50 000	1 (4.3)

*Note:* Data are displayed as either mean ± SD or *n* (%).

### 3.2 Feasibility

On average, parents took 14.8 min (SD = 4.7, range 6.2–28.4) to complete the pCMT‐QoL, while children took 11.5 min (SD = 3.6, range 4.2–16.2). Most parents and all children (82%) finished within 15 min. The remaining 18% of respondents, all of whom were parents with visual impairments, required assistance from a reader. Among the 18 children aged 8–18 years, child self‐reports were unavailable for 2 (8.7%) because of intellectual disabilities and behavioral problems, but their parents completed the parent‐proxy version instead. No data were missing from the parent reports.

### 3.3 Reliability

#### 3.3.1 Test–Retest Reliability

A subset of children (*n* = 21) and parents (*n* = 16) completed the pCMT‐QoL a second time by mail after 2–4 weeks. The parent‐proxy versions for ages 0–7 years and 8–18 years yielded ICCs of 0.90 and 0.91, respectively. The child version for ages 8–18 years had an ICC of 0.86. Domain‐specific ICCs ranged from 0.36 (social skills) to 0.88 (feelings). Notably, the social skills domain in the child version had lower reliability with an ICC of 0.36 (Table 3).

**TABLE 3:** Test–retest reliability of the Thai version of the pCMT‐QoL.

Domain	Age 0–7 (parent)		Age 8–18 (child)		Age 8–18 (parent)	
	(*N* = 5)		(*N* = 16)		(*N* = 16)	
	ICC	95% CI	ICC	95% CI	ICC	95% CI
Symptoms	0.84	(0.07, 0.98)	0.60	(0.17, 0.84)	0.71	(0.37, 0.88)
Function	0.90	(0.34, 0.99)	0.72	(0.36, 0.89)	0.90	(0.76, 0.96)
Social activities	0.99	(0.88, 1.00)	0.83	(0.59, 0.94)	0.79	(0.51, 0.91)
Feelings	0.89	(0.30, 0.99)	0.88	(0.70, 0.96)	0.88	(0.71, 0.95)
Cognition	0.65	(−0.34, 0.96)	0.82	(0.56, 0.93)	0.83	(0.60, 0.93)
Social skills	0.83	(0.05, 0.98)	0.36	(−0.15, 0.72)	0.73	(0.41, 0.89)
Physical domain score	0.96	(0.70, 0.99)	0.67	(0.28, 0.87)	0.74	(0.42, 0.89)
Mental domain score	0.84	(0.10, 0.98)	0.93	(0.81, 0.98)	0.88	(0.70, 0.95)
Total score	0.90	(0.34, 0.99)	0.86	(0.65, 0.95)	0.91	(0.79, 0.97)

#### 3.3.2 Internal Consistency

Internal consistency was evaluated using Cronbach's alpha at the first assessment (Table 4).

**TABLE 4:** Internal consistency of the Thai pCMT‐QoL across domains and age groups.

Domain	Standardized Cronbach's alpha		
	Ages 0–7 (parent) (*N* = 5)	Ages 8–18 (child) (*N* = 16)	Ages 8–18 (parent) (*N* = 18)
Physical: symptoms	0.85	0.79	0.57
Physical: function	0.79	0.66	0.89
Physical: social activities	0.83	0.61	0.88
Mental: feelings	0.75	0.89	0.95
Mental: cognition	0.83	0.78	0.92
Mental: social skills	0.78	0.73	0.83
Physical domain score	0.82	0.66	0.75
Mental domain score	0.79	0.89	0.96
Total score	0.83	0.88	0.91

The pCMT‐QoL exhibited strong internal consistency in most domains and age groups, with Cronbach's alpha values typically exceeding 0.70. Total scores for all groups showed high reliability, ranging from 0.83 to 0.91. However, three domains demonstrated lower reliability, including symptoms (*α* = 0.57) in the parent‐proxy version for ages 0–7 years, and social activities (*α* = 0.61) and function (*α* = 0.66) in the child self‐report for ages 8–18 years.

### 3.4 Parent–Child Agreement

Among children aged 8–18 years, parent‐proxy versus child self‐reported pCMT‐QoL scores differed across several domains (Table 5). Overall, the parent‐proxy total score (42.9 ± 14.5) was higher than the child self‐report total score (38.9 ± 10.6; *p* = 0.128), with a strong correlation (*r* = 0.72). In the physical domain, the parent score (53.8 ± 13.1) significantly exceeded the child score (44.0 ± 10.0; *p* = 0.006), showing a moderate correlation (*r* = 0.47). Conversely, in the mental domain, the child score (33.8 ± 14.4) was higher than the parent score (32.1 ± 23.1; *p* = 0.673).

**TABLE 5:** Comparison of parent‐proxy and child self‐reported pCMT‐QoL scores for ages 8–18 (*N* = 16).

Domain	Paired *t*‐test			Convergent validity	
	Child version score mean (SD)	Parent version score mean (SD)	*p*	Correlation (*r*)	*p*
Symptoms	34.6 (16.3)	61.8 (16.6)	< 0.001		
Function	43.9 (17.8)	50.7 (21.8)	0.175		
Social activities	53.4 (17.5)	48.7 (25.7)	0.432		
Feelings	41.9 (22.1)	38.8 (30.3)	0.640		
Cognition	25.8 (15.5)	31.4 (25.4)	0.256		
Social skills	33.6 (18.1)	26.1 (18.1)	0.102		
Physical domain score	44.0 (10.0)	53.8 (13.1)	0.006	0.47	0.063
Mental domain score	33.8 (14.4)	32.1 (23.1)	0.673	0.73	0.001
Total score	38.9 (10.6)	42.9 (14.5)	0.128	0.72	0.002

### 3.5 Convergent Validity

Pearson correlation analysis revealed strong convergence between parent‐proxy and child total scores as well as between mental composite domain scores. In contrast, the physical composite domain showed moderate correlation (Table 5). Additional analysis using the PedsQL 4.0 showed that total scores consistently demonstrated moderate to strong correlations across groups (Table 6).

**TABLE 6:** Correlation between pCMT‐QoL and PedsQL 4.0 across age groups.

pCMT‐QOL	PedsQL 4.0	Age 0–7 (parent) (*N* = 5)		Age 8–18 (child) (*N* = 16)		Age 8–18 (parent) (*N* = 18)	
		*r*	*p*	*r*	*p*	*r*	*p*
Symptoms	Physical functioning	0.93	0.023	0.10	0.715	−0.48	0.057
Function							
Social activities							
Feelings	Emotional functioning	0.49	0.398	0.70	0.004	0.76	< 0.001
Cognition	School functioning	0.29	0.638	0.56	0.031	0.52	0.040
Social skills	Social functioning	0.35	0.558	−0.15	0.591	0.47	0.063
Total score		0.59	0.292	0.55	0.036	0.78	< 0.001

*Note:* Gray‐shaded cells indicate domains not comparable between pCMT‐QoL and PedsQL 4.0. Hence, correlations were not calculated.

## 4 Discussion

We translated three original versions of the pCMT‐QoL instrument into Thai: parent‐proxy versions for ages 0–7 and 8–18 years, and a child version for ages 8–18 years [6, 8, 10]. The Thai pCMT‐QoL was then administered to 21 pediatric patients and 23 parents to assess its reliability and validity. No participants reported difficulties completing the questionnaire. The validated Thai version demonstrated high validity and reliability, suggesting strong potential for clinical and research use. It can effectively gauge health‐related QoL among pediatric CMT patients and identify areas for therapeutic improvement.

Test–retest reliability was high for all versions of the Thai pCMT‐QoL, with ICCs surpassing 0.85, which is consistent with findings from the English and Spanish versions of the pCMT‐QoL [7, 8, 9]. However, the lower ICC in the cognitive domain for the parent‐proxy version (ages 0–7) suggests variability in parental interpretations. This discrepancy may stem from caregivers' differing perceptions rather than actual changes in a child's condition. In future applications, providing brief guidance or instructions to caregivers may help standardize interpretations of symptoms and behaviors, reduce subjective differences, and improve the reliability of proxy assessments.

In addition, it was observed that scores in some domains, particularly Social Skills, fluctuated at the 2‐week follow‐up compared with baseline, resulting in lower ICC values. These changes likely reflect real‐life variability in children and adolescents with CMT—shaped by school attendance, peer interactions, family support, independence, and daily symptom fluctuations—rather than measurement error. Prior studies have similarly highlighted greater variability in psychosocial domains compared with physical functioning [22, 23].

Cronbach's alpha values showed high internal consistency across most domains, especially in the mental composite domain, which displayed robust reliability. However, the function and social activities domains for children aged 8–18 years exhibited notably low reliability, and similar concerns arose in the symptoms domain for the parent‐proxy version aged 0–7. These findings underscore the complexities of measuring QoL in pediatric populations, where rapid developmental changes, subjective interpretations, and varying parental observations can lead to inconsistent responses. Targeted modifications to these domains, along with including multiple informants, may help improve reliability.

Among children aged 8–18 years, parent‐proxy and child self‐reported pCMT‐QoL scores showed strong correlations for total and mental composite domains, confirming convergent validity. However, the physical composite domain correlated only moderately, reflecting divergent perceptions of physical limitations between children and parents. Parents also reported significantly higher total scores than children, a pattern consistent with the English version of the pCMT‐QoL [8] and other neuromuscular disease studies [24]. These discrepancies illustrate the importance of integrating both parent‐proxy and child self‐reports to capture a comprehensive view of the child's QoL. Indeed, research suggests that discordant responses between child and parent reports are common in health‐related QoL questionnaires, even outside chronic illness contexts [25, 26]. Thus, incorporating both perspectives remains crucial for accurately assessing pediatric well‐being.

This study confirms that the Thai version of the pCMT‐QoL instrument is culturally adapted, validated, and reliable, with performance comparable to the original English version. It also offers practical advantages, requiring about 15 min to complete and permitting remote administration. However, the small sample size—particularly in the 0–7‐year‐old parent‐proxy group—limits the generalizability of the findings, and the instrument's responsiveness to clinical changes over time remains untested. Given the rarity of pediatric CMT and the constrained sample, future research should involve larger and more diverse cohorts, along with its systematic use in clinical evaluations and follow‐up.

## 5 Conclusions

In summary, the Thai pCMT‐QoL is a reliable and valid measure for assessing the QoL of pediatric CMT patients in Thailand. Its comprehensive coverage of physical and mental health domains makes it a valuable resource for both patient‐centered care and research. Further validation in broader settings will maximize its clinical utility and ensure its adaptability across diverse populations.

## Author Contributions

**Pimchanok Kulsirichawaroj:** conceptualization, methodology, project administration, writing – original draft, visualization, funding acquisition. **Arisa Phochaisarn:** investigation. **Sivaporn Limpaninlachat:** methodology, formal analysis, validation, writing – review and editing. **Nutchavadee Vorasan:** formal analysis. **Surachai Likasitwattanakul:** resources, writing – review and editing. **Oranee Sanmaneechai:** conceptualization, methodology, resources, formal analysis, validation, writing – review and editing, supervision. **Michael E. Shy:** instrument development, writing – review and editing. **Sindhu Ramchandren:** instrument development, writing – review and editing.

## Conflicts of Interest

The authors declare no conflicts of interest.

## Supporting information


**Data S1:** Supporting Information.

**Data S2:** Supporting Information.

**Data S3:** Supporting Information.

## Data Availability

All relevant data supporting the findings of this study are included in the article and its Supporting Information [Link], [Link], [Link].

## Appendix A See Table A1

**TABLE A1:** English back‐translation of the Thai version of the pCMT‐QoL (parent‐proxy version for ages 0–7).

	Original English version	Backward translation
Symptoms		
1	Your child seems tired even with little activity	Your child seems exhausted even with minimal activity.
2	Your child complains often of pain	Your child often complains of pain.
3	Your child has difficulty doing things because of pain	Your child has difficulty doing things because of pain.
4	Your child has pain that lasts for a long time	Your child has pain for a long period of time.
5	Your child complains of leg or hand cramps	Your child complains of leg or hand cramps.
6	Your child complains of shaking (tremor) in the hands or legs	Your child complains of tremors in his/her hands or legs.
7	Your child seems very sleepy during the day	Your child looks very sleepy during the day.
8	Your child cannot do things they want to because they are tired	Your child cannot do what he/she wants to do because he/she is exhausted.
9	Your child cannot do things they want to because they are in pain	Your child cannot do what he/she wants to do because he/she is having pain.
10	Your child often trips and falls	Your child often trips over and falls.
11	Your child has had trouble with their balance while walking	Your child has difficulty balancing while walking.
12	Your child has tremor (shaking) of the arms or legs that interferes with physical activities	Your child has tremors in his/her arms or legs that interfere with his/her activities.
Function		
13	Your child can stand on their tip toes	Your child can stand on tip toes.
14	Your child can bend over without losing their balance	Your child can lean forward without losing balance.
15	Your child is able to twist open a jar lid by themselves	Your child can twist and open the screw cap by him/herself.
16	Your child is able to hold a plate full of food (using one or 2 hands) without spilling	Your child can hold a plate full of food (with one or two hands) without spilling.
17	Your child is able to put on shoes (any type) all by themselves	Your child can put on the shoes (any type) by him/herself.
18	Your child is able to zip up or button their clothes all by themselves	Your child can zip up or button up clothes by him/herself.
19	Your child is able to use a pen or pencil easily to write or scribble	Your child can easily use a pen or pencil to write or scribble.
20	Your child can get in and out of a bus or car easily	Your child can easily get on and off the bus or car.
21	Your child can keep up with the family when you all go out together	Your child can keep up with the family when going out together.
22	Your child is able to do their daily activities without asking for help	Your child can do daily activities without asking for help.
Social activities		
23	Your child enjoys playing with friends	Your child is happy to play with friends.
24	Your child can keep up with their friends during physical activities	Your child can keep up with his/her friends in any activity.
25	Your child spends a lot of time at home instead of outside because of CMT	Your child spends a lot of time at home instead of going out because of CMT.
26	Your child tires out faster than others during physical activity	Your child gets exhausted more easily than others during activities.
27	Your child is excluded when their friends play together	Your child is separated while his/her friends play together.
28	Your child avoids any task that involves physical activity	Your child avoids doing anything that requires physical activity.
29	Your child likes to be by themselves rather than with a group of friends	Your child prefers to be alone rather than with a group of friends.
Feelings		
30	Your child seems frustrated because of their CMT	Your child seems frustrated because of CMT.
31	Your child seems lonely	Your child seems lonely.
32	Your child seems sad	Your child seems sad.
33	Your child seems angry	Your child seems angry.
34	Your child seems anxious	Your child seems worried.
35	Your child seems unhappy about how they look	Your child seems unhappy with his/her self‐image.
36	People made fun of your child's feet or hands	Others make fun of your child's feet or hands.
37	Your child threw excessive tantrums	Your child shows excessive tantrums.
38	Your child followed your directions	Your child follows your instructions.
39	Your child has problems with anger management	Your child has problem with anger management.
Cognition		
40	Your child finds it hard to concentrate	Concentrating is difficult for your child.
41	Your child forgets tasks that they need to do	Your child forgets tasks that need to be done.
42	Your child has to read something several times to understand it	Your child must read something several times to understand it.
43	Your child has trouble paying attention	Your child has trouble giving attention.
44	Your child reads slower than other kids	Your child read slower than other children.
45	Your child has difficulty with finding the right words in a conversation	Your child has difficulty finding the right words in conversation.
46	Your child has a hard time keeping track of their work	Your child has trouble keeping up with his/her own tasks.
47	People have trouble understanding your child when they talk	Others find it difficult to understand your child when they are having conversations.
48	Your child has more trouble completing their work on time than other kids	Your child takes more time than other children to complete tasks.
49	Your child gets easily frustrated with their reading or writing projects	Your child is easily frustrated with reading or writing tasks.
Social skills		
50	Your child appears confident when with their friends	Your child seems confident when being with friends.
51	Your child appears confident when with adults	Your child seems confident when being with adults.
52	Your child is well‐behaved when outdoors	Your child is well behaved when going out.
53	Your child gets along well with the family	Your child gets along well with family members.
54	Your child seems happy at home	Your child looks happy at home.
55	Your child gets along well with their friends	Your child gets along well with friends.
56	Your child is able to express their needs easily	Your child can easily express his/her needs.
57	Your child takes pride in being independent	Your child is proud of his/her independence, not having to depend on others.

## Appendix B See Table B1

**TABLE B1:** English back‐translation of the Thai version of the pCMT‐QoL (parent‐proxy version for ages 8–18).

	Original English version	Backward translation
Symptoms		
1	Your child seems tired even with little activity	Your child seems exhausted even with minimal activity.
2	Your child complains often of pain	Your child often complains of pain.
3	Your child has difficulty doing things because of pain	Your child has difficulty doing things because of pain.
4	Your child has pain that lasts for a long time	Your child has pain for a long period of time.
5	Your child complains of leg or hand cramps	Your child complains of leg or hand cramps.
6	Your child complains of shaking (tremor) in the hands or legs	Your child complains of tremors in his/her hands or legs.
7	Your child seems very sleepy during the day	Your child looks very sleepy during the day.
8	Your child cannot do things they want to because they are tired	Your child is unable to do what he/she wants because he/she is exhausted.
9	Your child cannot do things they want to because they are in pain	Your child is unable to do what he/she wants because of the pain.
10	Your child often trips and falls	Your child always trips over and falls.
11	Your child has had trouble with their balance while walking	Your child has difficulty balancing while walking.
12	Your child has tremor (shaking) of the arms or legs that interferes with physical activities	Your child has tremors in his/her arms or legs that interfere with physical activities.
Function		
13	Your child can stand on their tip toes	Your child can stand on tip toes.
14	Your child can bend over without losing their balance	Your child can lean forward without losing balance.
15	Your child is able to twist open a jar lid by themselves	Your child can twist and open the screw cap by him/herself.
16	Your child is able to hold a plate full of food (using one or 2 hands) without spilling	Your child can hold a plate full of food (with one or two hands) without spilling.
17	Your child is able to put on shoes (any type) all by themselves	Your child can put on the shoes (any type) by him/herself.
18	Your child is able to zip up or button their clothes all by themselves	Your child can zip up or button up his/her shirt by him/herself.
19	Your child is able to use a pen or pencil easily to write	Your child can easily use a pen or pencil to write.
20	Your child can get in and out of a bus or car easily	Your child can easily get on and off the bus or car.
21	Your child can keep up with the family when you all go out together	Your child can keep up with the family when going out together.
22	Your child is able to do their daily activities without asking for help	Your child can do daily activities without asking for help.
Social activities		
23	Your child enjoys playing with friends	Your child is happy to play with friends.
24	Your child can keep up with their friends during physical activities	Your child can keep up with his/her friends in physical activity.
25	Your child spends a lot of time at home instead of outside because of CMT	Your child spends a lot of time at home instead of going out because of CMT.
26	Your child tires out faster than others during physical activity	Your child gets exhausted more easily than others during physical activities.
27	Your child is excluded when their friends plan physical activities	Your child is separated while friends plan to do physical activities together.
28	Your child avoids any task that involves physical activity	Your child avoids doing anything that requires physical activity.
29	Your child likes to be by themselves rather than with a group of friends	Your child prefers to be alone rather than with a group of friends.
Feelings		
30	Your child seems frustrated because of their CMT	Your child seems frustrated because of CMT.
31	Your child seems lonely	Your child seems lonely.
32	Your child seems sad	Your child seems sad.
33	Your child seems angry	Your child seems angry.
34	Your child seems anxious	Your child seems worried.
35	Your child seems unhappy about how they look	Your child seems unhappy with his/her self‐image.
36	People made fun of your child's feet or hands	Others make fun of your child's feet or hands.
37	Your child worries that their health will get worse in the future	Your child is worried that his/her health will get worse in the future.
38	Your child seems bothered by having to depend on others to help them	Your child seems displeased with the need for help from others.
39	Your child has problems with anger management	Your child has problem with anger management.
Cognition		
40	Your child finds it hard to concentrate	Concentrating is difficult for your child.
41	Your child forgets tasks that they need to do	Your child forgets tasks that need to be done.
42	Your child has to read something several times to understand it	Your child must read something several times to understand it.
43	Your child has trouble paying attention	Your child has trouble giving attention.
44	Your child reads slower than other kids	Your child reads more slowly than other children.
45	Your child has difficulty with finding the right words in a conversation	Your child has difficulty finding the right words in conversation.
46	Your child has a hard time keeping track of their work	Your child has trouble keeping up with his/her own tasks.
47	People have trouble understanding your child when they talk	Others find it difficult to understand your child when they are having conversations.
48	Your child has more trouble completing their work on time than other kids	Your child takes more time than other children to complete tasks.
49	Your child gets easily frustrated with their reading or writing projects	Your child is easily frustrated with reading or writing assignments.
Social skills		
50	Your child appears confident when with their friends	Your child seems confident when being with friends.
51	Your child appears confident when with adults	Your child seems confident when being with adults.
52	Your child is well‐behaved when outdoors	Your child is well behaved when going out.
53	Your child gets along well with the family	Your child gets along well with family members.
54	Your child seems happy at home	Your child looks happy at home.
55	Your child gets along well with their friends	Your child gets along well with friends.
56	Your child seems comfortable expressing their opinions	Your child seems comfortable in expressing his/her opinions.
57	Your child takes pride in being independent	Your child is proud of his/her independence, not having to depend on others.

## Appendix C See Table C1

**TABLE C1:** English back‐translation of the Thai version of the pCMT‐QoL (child version for ages 8–18).

	Original English version	Backward translation
Symptoms		
1	I feel tired even with little activity	I feel exhausted even with little activity
2	I often have pain	I often have pain.
3	I have difficulty doing things because of pain	I have difficulty doing things because of the pain.
4	I have pain that lasts for a long time	I have pain for a long period of time.
5	I have had leg or hand cramps	I have leg or hand cramps.
6	I have shaking (tremor) in my hands or legs	I have tremors in my hands or legs.
7	I am very sleepy during the day	I feel very sleepy during the day.
8	I cannot do things I want to because I am tired	I cannot do what I want because I am exhausted.
9	I cannot do things I want to because I am in pain	I cannot do what I want because of the pain I have.
10	I often trip and fall	I often trip over and fall.
11	I have had trouble with my balance while walking	I have difficulty balancing while walking.
12	I have tremor (shaking) of the arms or legs that keep me from being physically active	I have tremors in my arms or legs that interfere with my physical activity.
Function		
13	I can stand on my tip toes	I can stand on my tip toes.
14	I can bend over without losing my balance	I can lean forward without losing balance.
15	I am able to twist open a jar lid by myself	I can twist and open the screw cap by myself.
16	I am able to hold a plate full of food (using one or 2 hands) without spilling	I can hold a plate full of food (with one or two hands) without spilling.
17	I am able to put on shoes (any type) all by myself	I can put on the shoes (any type) by myself.
18	I am able to zip up or button my clothes all by myself	I can zip up or button up the shirt by myself.
19	I am able to use a pen or pencil easily to write	I can easily use a pen or pencil to write.
20	I can get in and out of a bus or car easily	I can easily get on and off the bus or car.
21	I can keep up with the family when we all go out together	I can keep up with the family when going out together.
22	I am able to do my daily activities without asking for help	I can do daily activities without asking for help from others.
Social activities		
23	I enjoy physical activities with friends	I am happy to play with friends.
24	I can keep up with my friends during physical activities	I can keep up with my friends in physical activity.
25	I spend a lot of time at home instead of outside because of my CMT	I spend a lot of time at home instead of going out because of CMT.
26	I tire out faster than others during physical activity	I get exhausted more easily than others during physical activities.
27	I am excluded when my friends plan physical activities	I am separated while friends plan to do physical activities together.
28	I avoid any task that involves physical activity	I avoid doing anything that requires physical activity.
29	I like to be by myself rather than with a group of friends	I prefer to be alone rather than with a group of friends.
Feelings		
30	I feel frustrated because of my CMT	I feel frustrated because of the CMT I have.
31	I feel lonely because of my CMT	I feel lonely because of the CMT I have.
32	I feel sad because of my CMT	I feel sad because of the CMT I have.
33	I feel angry because of my CMT	I feel angry because of the CMT I have.
34	I feel anxious because of my CMT	I feel worried because of the CMT I have.
35	I am unhappy about how I look	I am unhappy with my self‐image.
36	People made fun of my feet or hands	Others make fun of my feet or hands.
37	I worry that my health will get worse in the future	I am worried that my health will get worse in the future.
38	I am bothered by having to depend on others to help me	I don't like having to rely on other people's help.
39	I have problems with my anger	I have problem with anger management.
Cognition		
40	I find it hard to concentrate	Concentrating is difficult for me.
41	I forget things that I need to do	I forget tasks that need to be done.
42	I have to read something several times to understand it	I must read something several times to understand it.
43	I have trouble paying attention	I have trouble giving attention.
44	I read slower than friends my age	I read slower than other children at the same age.
45	I have difficulty with finding the right words when I am talking to someone	I have difficulty finding the right words in conversation.
46	I have a hard time keeping track of my work	I have trouble keeping up with my own tasks.
47	People have trouble understanding me when I talk	Others find it difficult to understand me when we are having conversations.
48	I have more trouble completing my work on time than my friends	I take longer to submit my own tasks on time when comparing to other children.
49	I get easily frustrated with my reading or writing projects	I am easily frustrated with reading or writing assignments.
Social skills		
50	I feel confident when I am with my friends	I feel confident when being with friends.
51	I feel confident when I am with adults	I feel confident when being with adults.
52	I am well‐behaved when outdoors	I am well behaved when going out.
53	I get along well with the family	I get along well with my family members.
54	I am happy at home	I am happy at home
55	I get along well with my friends	I get along well with friends.
56	I am comfortable talking about my opinions (how I feel about something)	I feel comfortable expressing my opinions (about my feelings on things).
57	I take pride in being independent	I am proud of being independent and requiring no help from others, not having to depend on others.

## References

[1] M. M. Reilly , S. M. Murphy , and M. Laurá , “Charcot‐Marie‐Tooth Disease,” Journal of the Peripheral Nervous System 16, no. 1 (2011): 1–14, 10.1111/j.1529-8027.2011.00324.x. 21504497

[2] L. Benarroch , G. Bonne , F. Rivier , and D. Hamroun , “The 2024 Version of the Gene Table of Neuromuscular Disorders (Nuclear Genome),” Neuromuscular Disorders 34 (2024): 126–170, 10.1016/j.nmd.2023.12.007. 38253411

[3] A. M. Rossor , M. E. Shy , and M. M. Reilly , “Are We Prepared for Clinical Trials in Charcot‐Marie‐Tooth Disease?,” Brain Research 1729 (2020): 146625, 10.1016/j.brainres.2019.146625. 31899213 PMC8418667

[4] J. Burns , R. Ouvrier , T. Estilow , et al., “Validation of the Charcot‐Marie‐Tooth Disease Pediatric Scale as an Outcome Measure of Disability,” Annals of Neurology 71, no. 5 (2012): 642–652, 10.1002/ana.23572. 22522479 PMC3335189

[5] M. R. Mandarakas , M. P. Menezes , K. J. Rose , et al., “Development and Validation of the Charcot‐Marie‐Tooth Disease Infant Scale,” Brain 141, no. 12 (2018): 3319–3330, 10.1093/brain/awy280. 30476010 PMC6312041

[6] S. Ramchandren , T. T. Wu , R. S. Finkel , et al., “Development and Validation of the Pediatric Charcot‐Marie‐Tooth Disease Quality of Life Outcome Measure,” Annals of Neurology 89, no. 2 (2021): 369–379, 10.1002/ana.25966. 33222249 PMC11671102

[7] I. Moroni , F. R. Danti , D. Pareyson , et al., “Validation of the Italian Version of the Pediatric CMT Quality of Life Outcome Measure,” Journal of the Peripheral Nervous System 27, no. 2 (2022): 127–130, 10.1111/jns.12494. 35416371 PMC9324941

[8] T. T. Wu , R. S. Finkel , C. E. Siskind , et al., “Validation of the Parent‐Proxy Pediatric Charcot‐Marie‐Tooth Disease Quality of Life Outcome Measure,” Journal of the Peripheral Nervous System 28, no. 2 (2023): 237–251, 10.1111/jns.12538. 36748295 PMC10521146

[9] F. R. Danti , E. Pagliano , D. Pareyson , et al., “Parent‐Proxy Pediatric CMT Quality of Life Outcome Measure: Validation of the Italian Version,” Journal of the Peripheral Nervous System 29, no. 1 (2024): 107–110, 10.1111/jns.12615. 38329138

[10] T. T. Wu , R. S. Finkel , C. E. Siskind , et al., “Validation of the Parent‐Proxy Version of the Pediatric Charcot‐Marie‐Tooth Disease Quality of Life Instrument for Children Aged 0‐7 Years,” Journal of the Peripheral Nervous System 28, no. 3 (2023): 382–389, 10.1111/jns.12557. 37166413

[11] P. Kulsirichawaroj , Y. Suksangkharn , D. E. Nam , et al., “Gene Distribution in Pediatric‐Onset Inherited Peripheral Neuropathy: A Single Tertiary Center in Thailand,” Journal of Neuromuscular Diseases 11, no. 1 (2024): 191–199, 10.3233/jnd-230174. 37927275 PMC10789325

[12] A. Thongsing , T. Pho‐Iam , C. Limwongse , S. Likasitwattanakul , and O. Sanmaneechai , “Case Series: Childhood Charcot‐Marie‐Tooth: Predominance of Axonal Subtype,” eNeurologicalSci 16 (2019): 100200, 10.1016/j.ensci.2019.100200. 31417964 PMC6690715

[13] D. Wild , A. Grove , M. Martin , et al., “Principles of Good Practice for the Translation and Cultural Adaptation Process for Patient‐Reported Outcomes (PRO) Measures: Report of the ISPOR Task Force for Translation and Cultural Adaptation,” Value in Health 8, no. 2 (2005): 94–104, 10.1111/j.1524-4733.2005.04054.x. 15804318

[14] J. Borsa , B. Damasio , and D. Bandeira , “Cross‐Cultural Adaptation and Validation of Psychological Instruments: Some Considerations,” Paidéia (Ribeirão Preto) 22 (2012): 10.

[15] J. W. Varni , M. Seid , and P. S. Kurtin , “PedsQL 4.0: Reliability and Validity of the Pediatric Quality of Life Inventory Version 4.0 Generic Core Scales in Healthy and Patient Populations,” Medical Care 39, no. 8 (2001): 800–812, 10.1097/00005650-200108000-00006. 11468499

[16] P. Sritipsukho , M. Wisai , and M. Thavorncharoensap , “Reliability and Validity of the Thai Version of the Pediatric Quality of Life Inventory 4.0,” Quality of Life Research 22, no. 3 (2013): 551–557, 10.1007/s11136-012-0190-y. 22552603

[17] P. A. Harris , R. Taylor , R. Thielke , J. Payne , N. Gonzalez , and J. G. Conde , “Research Electronic Data Capture (REDCap)—A Metadata‐Driven Methodology and Workflow Process for Providing Translational Research Informatics Support,” Journal of Biomedical Informatics 42, no. 2 (2009): 377–381, 10.1016/j.jbi.2008.08.010. 18929686 PMC2700030

[18] T. K. Koo and M. Y. Li , “A Guideline of Selecting and Reporting Intraclass Correlation Coefficients for Reliability Research,” Journal of Chiropractic Medicine 15, no. 2 (2016): 155–163, 10.1016/j.jcm.2016.02.012. 27330520 PMC4913118

[19] L. G. Portney and M. P. Watkins , Foundations of Clinical Research: Applications to Practice (Pearson/Prentice Hall, 2015).

[20] K. S. Taber , “The Use of Cronbach's Alpha When Developing and Reporting Research Instruments in Science Education,” Research in Science Education 48, no. 6 (2018): 1273–1296, 10.1007/s11165-016-9602-2.

[21] P. Schober , C. Boer , and L. A. Schwarte , “Correlation Coefficients: Appropriate Use and Interpretation,” Anesthesia and Analgesia 126, no. 5 (2018): 1763–1768. 29481436 10.1213/ANE.0000000000002864

[22] C. Eiser and R. Morse , “Quality‐Of‐Life Measures in Chronic Diseases of Childhood,” Health Technology Assessment 5, no. 4 (2001): 1–157, 10.3310/hta5040. 11262421

[23] J. W. Varni , C. A. Limbers , and T. M. Burwinkle , “How Young Can Children Reliably and Validly Self‐Report Their Health‐Related Quality of Life?: An Analysis of 8,591 Children Across Age Subgroups With the PedsQL 4.0 Generic Core Scales,” Health and Quality of Life Outcomes 5 (2007): 1, 10.1186/1477-7525-5-1. 17201920 PMC1769360

[24] C. Campbell , E. McColl , M. P. McDermott , W. B. Martens , M. Guglieri , and R. C. Griggs , “Health Related Quality of Life in Young, Steroid‐Naïve Boys With Duchenne Muscular Dystrophy,” Neuromuscular Disorders 31, no. 11 (2021): 1161–1168, 10.1016/j.nmd.2021.06.001. 34489153

[25] C. Eiser and R. Morse , “Can Parents Rate Their Child's Health‐Related Quality of Life? Results of a Systematic Review,” Quality of Life Research 10, no. 4 (2001): 347–357, 10.1023/a:1012253723272. 11763247

[26] P. Upton , C. Eiser , I. Cheung , et al., “Measurement Properties of the UK‐English Version of the Pediatric Quality of Life Inventory 4.0 (PedsQL) Generic Core Scales,” Health and Quality of Life Outcomes 3 (2005): 22, 10.1186/1477-7525-3-22. 15804349 PMC1079918

