# Supplementary material for: Validation and Reliability of the Thai Pediatric Charcot–Marie–Tooth Quality of Life Outcome Measure
Source: J Peripher Nerv Syst. 2025 Nov 16;30(4):e70075. doi: 10.1111/jns.70075 (PMC12620538; doi:10.1111/jns.70075)
Supplement: Supplementary file 1 — Data S1: Supporting Information. [file JNS-30-0-s003.pdf]

รหัส: ______________________________

โรงพยาบาลและคลินิก มหาวิทยาลัยไอโอวา

เครื่องมือวัดคุณภาพชีวิตสำหรับโรคเส้นประสาทจากพันธุกรรมซีเอ็มทีในเด็ก (pCMT-QOL)

ฉบับผู้ปกครอง (สำหรับเด็กอายุ 7 ปีหรือน้อยกว่า)

ผู้วิจัย: แพทย์หญิงสินธุ แรมจันเดรน (วท.บ.)

คำชี้แจงสำหรับพ่อแม่ / ผู้ปกครองตามกฎหมาย

โปรดตอบแบบสอบถามนี้ โดยให้ผู้ปกครองตอบแบบสอบถามนี้เพียงคนเดียว หากคุณมีลูกที่เป็นโรคเส้นประสาทจากพันธุกรรมซีเอ็มทีมากกว่าหนึ่งคน โปรดตอบแบบสอบถามแยกกันหนึ่งฉบับสำหรับลูกแต่ละคน

ในแบบสอบถามนี้ ไม่มีคำตอบที่ถูกหรือผิด โปรดเลือกเพียงหนึ่งคำตอบสำหรับแต่ละข้อคำถาม และโปรดตอบให้ครบทุกข้อ

รหัส: ______________________________

	แม่	พ่อ	ผู้ปกครองตามกฎหมาย	อื่นๆ
แบบสอบถามนี้ตอบโดย	☐	☐	☐	☐

<u>โปรดทำเครื่องหมายลงในช่องคำตอบเพียงช่องเดียวของแต่ละข้อความต่อไปนี้</u>

***อาการ*:**

	ระยะหลังมานี้	ไม่เคย	แทบไม่เคย	บางครั้ง	แทบทุกครั้ง	ทุกครั้ง
1	ลูกของคุณดูหมดแรงแม้จะทำกิจกรรมเพียงเล็กน้อย	☐ 0	☐ 1	☐ 2	☐ 3	☐ 4
2	ลูกของคุณมักบ่นว่าปวด	☐ 0	☐ 1	☐ 2	☐ 3	☐ 4
3	ลูกของคุณทำสิ่งต่าง ๆ ได้ยากเพราะปวด	☐ 0	☐ 1	☐ 2	☐ 3	☐ 4
4	ลูกของคุณมีอาการปวดที่ยาวนาน	☐ 0	☐ 1	☐ 2	☐ 3	☐ 4
5	ลูกของคุณบ่นว่าเป็นตะคริวที่ขาหรือมือ	☐ 0	☐ 1	☐ 2	☐ 3	☐ 4
6	ลูกของคุณบ่นว่ามีอาการสั่นที่มือหรือขา	☐ 0	☐ 1	☐ 2	☐ 3	☐ 4
7	ลูกของคุณดูง่วงนอนมากในเวลากลางวัน	☐ 0	☐ 1	☐ 2	☐ 3	☐ 4
8	ลูกของคุณไม่สามารถทำสิ่งที่เขาต้องการได้เพราะหมดแรง	☐ 0	☐ 1	☐ 2	☐ 3	☐ 4
9	ลูกของคุณไม่สามารถทำสิ่งที่เขาต้องการได้เพราะปวด	☐ 0	☐ 1	☐ 2	☐ 3	☐ 4
10	ลูกของคุณมักสะดุดและหกล้ม	☐ 0	☐ 1	☐ 2	☐ 3	☐ 4
11	ลูกของคุณทรงตัวได้ยากขณะเดิน	☐ 0	☐ 1	☐ 2	☐ 3	☐ 4
12	ลูกของคุณมีอาการแขนหรือขาสั่นซึ่งรบกวนการทำกิจกรรมทางกาย	☐ 0	☐ 1	☐ 2	☐ 3	☐ 4

รหัส: ____________________________

<u>โปรดทำเครื่องหมายลงในช่องคำตอบเพียงช่องเดียวของแต่ละข้อความต่อไปนี้</u>

<u>ความสามารถ:</u>

	ระยะหลังมานี้	ไม่เคย	แทบไม่เคย	บางครั้ง	แทบทุกครั้ง	ทุกครั้ง
13	ลูกของคุณสามารถยืนเขย่งปลายเท้าได้	☐ 4	☐ 3	☐ 2	☐ 1	☐ 0
14	ลูกของคุณสามารถโน้มตัวได้โดยไม่เสียการทรงตัว	☐ 4	☐ 3	☐ 2	☐ 1	☐ 0
15	ลูกของคุณสามารถบิดเปิดฝาขวดเกลียวได้ด้วยตัวเอง	☐ 4	☐ 3	☐ 2	☐ 1	☐ 0
16	ลูกของคุณสามารถถืออาหารเต็มจาน (ด้วยหนึ่งหรือสองมือ) โดยไม่หก	☐ 4	☐ 3	☐ 2	☐ 1	☐ 0
17	ลูกของคุณสามารถใส่รองเท้า (ชนิดใดก็ได้) ด้วยตัวเอง	☐ 4	☐ 3	☐ 2	☐ 1	☐ 0
18	ลูกของคุณสามารถรูดซิปหรือติดกระดุมเสื้อผ้า ได้ด้วยตัวเอง	☐ 4	☐ 3	☐ 2	☐ 1	☐ 0
19	ลูกของคุณสามารถใช้ปากกาหรือดินสอในการเขียน ได้โดยง่าย	☐ 4	☐ 3	☐ 2	☐ 1	☐ 0
20	ลูกของคุณสามารถขึ้น-ลงรถโดยสารประจำทาง หรือรถยนต์ได้โดยง่าย	☐ 4	☐ 3	☐ 2	☐ 1	☐ 0
21	ลูกของคุณสามารถเดินตามครอบครัวได้ทัน เมื่อไปข้างนอกด้วยกัน	☐ 4	☐ 3	☐ 2	☐ 1	☐ 0
22	ลูกของคุณสามารถทำกิจวัตรประจำวันได้ โดยไม่ต้องขอความช่วยเหลือ	☐ 4	☐ 3	☐ 2	☐ 1	☐ 0

รหัส: ______________________________

<u>โปรดทำเครื่องหมายลงในช่องคำตอบเพียงช่องเดียวของแต่ละข้อความต่อไปนี้</u>

***<u>กิจกรรมทางสังคม</u>*:**

	ระยะหลังมานี้	ไม่เคย	แทบไม่เคย	บางครั้ง	แทบทุกครั้ง	ทุกครั้ง
23	ลูกของคุณสนุกในการเล่นกับเพื่อน ๆ	☐ 4	☐ 3	☐ 2	☐ 1	☐ 0
24	ลูกของคุณสามารถทำกิจกรรมทางกายได้ทันเพื่อน ๆ	☐ 4	☐ 3	☐ 2	☐ 1	☐ 0
25	ลูกของคุณใช้เวลาที่บ้านมาก แทนที่จะไปข้างนอกเพราะโรคซีเอ็มที	☐ 0	☐ 1	☐ 2	☐ 3	☐ 4
26	ลูกของคุณหมดแรงง่ายกว่าเพื่อนวัยเดียวกัน ขณะทำกิจกรรมทางกาย	☐ 0	☐ 1	☐ 2	☐ 3	☐ 4
27	ลูกของคุณถูกแยกออกมาขณะที่เพื่อน ๆ วางแผนจะทำกิจกรรมทางกายร่วมกัน	☐ 0	☐ 1	☐ 2	☐ 3	☐ 4
28	ลูกของคุณเลี่ยงที่จะทำสิ่งใด ๆ ก็ตามที่ต้องเคลื่อนไหวร่างกาย	☐ 0	☐ 1	☐ 2	☐ 3	☐ 4
29	ลูกของคุณชอบที่จะอยู่คนเดียวมากกว่า อยู่กับกลุ่มเพื่อน	☐ 0	☐ 1	☐ 2	☐ 3	☐ 4

รหัส: ______________________________

<u>โปรดทำเครื่องหมายลงในช่องคำตอบเพียงช่องเดียวของแต่ละข้อความต่อไปนี้</u>

<u>ความรู้สึก:</u>

	ระยะหลังมานี้	ไม่เคย	แทบไม่เคย	บางครั้ง	แทบทุกครั้ง	ทุกครั้ง
30	ลูกของคุณดูหงุดหงิดเนื่องจากโรคซีเอ็มที	☐ 0	☐ 1	☐ 2	☐ 3	☐ 4
31	ลูกของคุณดูเหงา	☐ 0	☐ 1	☐ 2	☐ 3	☐ 4
32	ลูกของคุณดูเศร้า	☐ 0	☐ 1	☐ 2	☐ 3	☐ 4
33	ลูกของคุณดูโกรธ	☐ 0	☐ 1	☐ 2	☐ 3	☐ 4
34	ลูกของคุณดูวิตกกังวล	☐ 0	☐ 1	☐ 2	☐ 3	☐ 4
35	ลูกของคุณดูไม่มีความสุขกับภาพลักษณ์ของตัวเอง	☐ 0	☐ 1	☐ 2	☐ 3	☐ 4
36	คนอื่นๆ ล้อเลียนเรื่องเท้าหรือมือของลูกของคุณ	☐ 0	☐ 1	☐ 2	☐ 3	☐ 4
37	ลูกของคุณแสดงอารมณ์ฉุนเฉียวอย่างเกินเหตุ	☐ 0	☐ 1	☐ 2	☐ 3	☐ 4
38	ลูกของคุณปฏิบัติตามคำแนะนำของคุณ	☐ 0	☐ 1	☐ 2	☐ 3	☐ 4
39	ลูกของคุณมีปัญหาในการจัดการอารมณ์โกรธ	☐ 0	☐ 1	☐ 2	☐ 3	☐ 4

รหัส: ______________________________

<u>โปรดทำเครื่องหมายลงในช่องคำตอบเพียงช่องเดียวของแต่ละข้อความต่อไปนี้</u>

<u>การรู้คิด:</u>

	ระยะหลังมานี้	ไม่เคย	แทบไม่เคย	บางครั้ง	แทบทุกครั้ง	ทุกครั้ง
40	การมีสมาธิเป็นเรื่องยากสำหรับลูกของคุณ	☐ 0	☐ 1	☐ 2	☐ 3	☐ 4
41	ลูกของคุณลืมงานที่จำเป็นต้องทำ	☐ 0	☐ 1	☐ 2	☐ 3	☐ 4
42	ลูกของคุณต้องอ่านบางอย่างหลายครั้งกว่าจะเข้าใจ	☐ 0	☐ 1	☐ 2	☐ 3	☐ 4
43	ลูกของคุณมีปัญหาในการให้ความตั้งใจ	☐ 0	☐ 1	☐ 2	☐ 3	☐ 4
44	ลูกของคุณอ่านได้ช้ากว่าเด็กคนอื่น ๆ	☐ 0	☐ 1	☐ 2	☐ 3	☐ 4
45	ลูกของคุณมีปัญหาในการหาคำที่เหมาะสมในการสนทนา	☐ 0	☐ 1	☐ 2	☐ 3	☐ 4
46	ลูกของคุณมีปัญหาในการติดตามงานของตนเอง	☐ 0	☐ 1	☐ 2	☐ 3	☐ 4
47	คนอื่นเข้าใจลูกของคุณได้ยากเมื่อคุยกัน	☐ 0	☐ 1	☐ 2	☐ 3	☐ 4
48	ลูกของคุณทำงานให้เสร็จทันเวลาได้ช้ากว่าเด็กคนอื่น ๆ	☐ 0	☐ 1	☐ 2	☐ 3	☐ 4
49	ลูกของคุณหงุดหงิดง่ายกับงานอ่านหรือเขียนที่ได้รับมอบหมาย	☐ 0	☐ 1	☐ 2	☐ 3	☐ 4

รหัส: ______________________________

<u>โปรดทำเครื่องหมายลงในช่องคำตอบเพียงช่องเดียวของแต่ละข้อความต่อไปนี้</u>

***ทักษะทางสังคม*:**

	ระยะหลังมานี้	ไม่เคย	แทบไม่เคย	บางครั้ง	แทบทุกครั้ง	ทุกครั้ง
5 0	ลูกของคุณดูมั่นใจเมื่ออยู่กับเพื่อน	☐ 4	☐ 3	☐ 2	☐ 1	☐ 0
51	ลูกของคุณดูมั่นใจเมื่ออยู่กับผู้ใหญ่	☐ 4	☐ 3	☐ 2	☐ 1	☐ 0
52	ลูกของคุณมีความประพฤติดีเมื่ออยู่นอกบ้าน	☐ 4	☐ 3	☐ 2	☐ 1	☐ 0
53	ลูกของคุณเข้ากันได้ดีกับคนในครอบครัว	☐ 4	☐ 3	☐ 2	☐ 1	☐ 0
54	ลูกของคุณดูมีความสุขเมื่ออยู่บ้าน	☐ 4	☐ 3	☐ 2	☐ 1	☐ 0
55	ลูกของคุณเข้ากับเพื่อนได้ดี	☐ 4	☐ 3	☐ 2	☐ 1	☐ 0
56	ลูกของคุณดูสบายใจที่ได้แสดงความคิดเห็นของตนเอง	☐ 4	☐ 3	☐ 2	☐ 1	☐ 0
57	ลูกของคุณภูมิใจในความมีอิสระ ไม่ต้องพึ่งผู้อื่น	☐ 4	☐ 3	☐ 2	☐ 1	☐ 0
