# Supplementary material for: Validation and Reliability of the Thai Pediatric Charcot–Marie–Tooth Quality of Life Outcome Measure
Source: J Peripher Nerv Syst. 2025 Nov 16;30(4):e70075. doi: 10.1111/jns.70075 (PMC12620538; doi:10.1111/jns.70075)
Supplement: Supplementary file 3 — Data S3: Supporting Information. [file JNS-30-0-s002.pdf]

รหัส: ____________________________

โรงพยาบาลและคลินิก มหาวิทยาลัยไอโอวา

เครื่องมือวัดคุณภาพชีวิตสำหรับโรคเส้นประสาท จากพันธุกรรมซีเอ็มทีในเด็ก (pCMT-QOL)

ฉบับเด็ก (สำหรับเด็กอายุ 8-18 ปี)

ผู้วิจัย: นายแพทย์ไมเคิล อี. ชาย (วท.บ.)

<u>คำชี้แจงสำหรับพ่อแม่ / ผู้ปกครองตามกฎหมาย</u>

โปรดตอบแบบสอบถามนี้ โดยให้ผู้ปกครองตอบแบบสอบถามนี้เพียงคนเดียว หากคุณมีลูกที่เป็นโรคเส้นประสาทจากพันธุกรรมซีเอ็มทีมากกว่าหนึ่งคน โปรดตอบแบบสอบถามแยกกันหนึ่งฉบับสำหรับลูกแต่ละคน

ในแบบสอบถามนี้ ไม่มีคำตอบที่ถูกหรือผิด โปรดเลือกเพียงหนึ่งคำตอบสำหรับแต่ละข้อคำถาม และโปรดตอบให้ครบทุกข้อ

มีแบบสอบถามแยกต่างหากสำหรับเด็กซึ่งเด็กต้องตอบ เราต้องการให้พวกเขาตอบคำถามเหล่านั้นด้วยตัวเอง แต่ถ้าพวกเขามีปัญหาในการถือแบบสอบถามหรือดินสอ คุณอาจช่วยวงกลมคำตอบที่ลูกเลือกได้

รหัส: ____________________________

	แม่	พ่อ	ผู้ปกครองตามกฎหมาย	อื่นๆ
แบบสอบถามนี้ตอบโดย	☐	☐	☐	☐

<u>โปรดทำเครื่องหมายลงในช่องคำตอบเพียงช่องเดียวของแต่ละข้อความต่อไปนี้</u>

<u>อาการ</u>:

	ระยะหลังมานี้	ไม่เคย	แทบไม่เคย	บางครั้ง	แทบทุกครั้ง	ทุกครั้ง
1	หนูรู้สึกหมดแรงแม้จะทำกิจกรรมเพียงเล็กน้อย	☐ 0	☐ 1	☐ 2	☐ 3	☐ 4
2	หนูมักมีอาการปวด	☐ 0	☐ 1	☐ 2	☐ 3	☐ 4
3	หนูทำสิ่งต่าง ๆ ได้ยากเพราะปวด	☐ 0	☐ 1	☐ 2	☐ 3	☐ 4
4	หนูมีอาการปวดที่ยาวนาน	☐ 0	☐ 1	☐ 2	☐ 3	☐ 4
5	หนูเป็นตะคริวที่ขาหรือมือ	☐ 0	☐ 1	☐ 2	☐ 3	☐ 4
6	หนูมีอาการสั่นที่มือหรือขา	☐ 0	☐ 1	☐ 2	☐ 3	☐ 4
7	หนูง่วงนอนมากในเวลากลางวัน	☐ 0	☐ 1	☐ 2	☐ 3	☐ 4
8	หนูไม่สามารถทำสิ่งที่ต้องการได้เพราะหมดแรง	☐ 0	☐ 1	☐ 2	☐ 3	☐ 4
9	หนูไม่สามารถทำสิ่งที่ต้องการได้เพราะปวด	☐ 0	☐ 1	☐ 2	☐ 3	☐ 4
10	หนูมักสะดุดและหกล้ม	☐ 0	☐ 1	☐ 2	☐ 3	☐ 4
11	หนูทรงตัวได้ยากขณะเดิน	☐ 0	☐ 1	☐ 2	☐ 3	☐ 4
12	หนูมีอาการแขนหรือขาสั่นทำให้ไม่อยากทำกิจกรรมทางกาย	☐ 0	☐ 1	☐ 2	☐ 3	☐ 4

รหัส: ______________________________

<u>โปรดทำเครื่องหมายลงในช่องคำตอบเพียงช่องเดียวของแต่ละข้อความต่อไปนี้</u>

<u>ความสามารถ:</u>

	ระยะหลังมานี้	ไม่เคย	แทบไม่เคย	บางครั้ง	แทบทุกครั้ง	ทุกครั้ง
13	หนูสามารถยืนเขย่งปลายเท้าได้	☐ 4	☐ 3	☐ 2	☐ 1	☐ 0
14	หนูสามารถโน้มตัวได้โดยไม่เสียการทรงตัว	☐ 4	☐ 3	☐ 2	☐ 1	☐ 0
15	หนูสามารถบิดเปิดฝาขวดเกลียวเองได้ด้วยตัวเอง	☐ 4	☐ 3	☐ 2	☐ 1	☐ 0
16	หนูสามารถถืออาหารเต็มจาน (ด้วยหนึ่งหรือสองมือ) โดยไม่หก	☐ 4	☐ 3	☐ 2	☐ 1	☐ 0
17	หนูสามารถใส่รองเท้า (ชนิดใดก็ได้) ด้วยตัวเอง	☐ 4	☐ 3	☐ 2	☐ 1	☐ 0
18	หนูสามารถรูดซิปหรือติดกระดุมเสื้อผ้าได้ด้วยตัวเอง	☐ 4	☐ 3	☐ 2	☐ 1	☐ 0
19	หนูสามารถใช้ปากกาหรือดินสอในการเขียนได้โดยง่าย	☐ 4	☐ 3	☐ 2	☐ 1	☐ 0
20	หนูสามารถขึ้น-ลงรถโดยสารประจำทางหรือรถยนต์ได้โดยง่าย	☐ 4	☐ 3	☐ 2	☐ 1	☐ 0
21	หนูสามารถเดินตามครอบครัวได้ทันเมื่อไปข้างนอกด้วยกัน	☐ 4	☐ 3	☐ 2	☐ 1	☐ 0
22	หนูทำกิจวัตรประจำวันได้โดยไม่ต้องขอความช่วยเหลือ	☐ 4	☐ 3	☐ 2	☐ 1	☐ 0

รหัส: ______________________________

<u>โปรดทำเครื่องหมายลงในช่องคำตอบเพียงช่องเดียวของแต่ละข้อความต่อไปนี้</u>

***<u>กิจกรรมทางสังคม</u>*:**

	ระยะหลังมานี้	ไม่เคย	แทบไม่เคย	บางครั้ง	แทบทุกครั้ง	ทุกครั้ง
23	หนูสนุกกับการทำกิจกรรมทางกายกับเพื่อน ๆ	☐ 4	☐ 3	☐ 2	☐ 1	☐ 0
24	หนูสามารถทำกิจกรรมทางกายได้ทันเพื่อน ๆ	☐ 4	☐ 3	☐ 2	☐ 1	☐ 0
25	หนูใช้เวลาที่บ้านมาก แทนที่จะไปข้างนอก เพราะโรคซีเอ็มที	☐ 0	☐ 1	☐ 2	☐ 3	☐ 4
26	หนูหมดแรงง่ายกว่าเพื่อนวัยเดียวกัน ขณะทำกิจกรรมทางกาย	☐ 0	☐ 1	☐ 2	☐ 3	☐ 4
27	หนูถูกแยกออกมาขณะที่เพื่อน ๆ วางแผนจะทำกิจกรรมทางกายร่วมกัน	☐ 0	☐ 1	☐ 2	☐ 3	☐ 4
28	หนูเลี่ยงที่จะทำสิ่งใด ๆ ก็ตามที่ต้องเคลื่อนไหวร่างกาย	☐ 0	☐ 1	☐ 2	☐ 3	☐ 4
29	หนูชอบที่จะอยู่คนเดียวมากกว่าอยู่กับกลุ่มเพื่อน	☐ 0	☐ 1	☐ 2	☐ 3	☐ 4

รหัส: ______________________________

<u>โปรดทำเครื่องหมายลงในช่องคำตอบเพียงช่องเดียวของแต่ละข้อความต่อไปนี้</u>

<u>ความรู้สึก:</u>

	ระยะหลังมานี้	ไม่เคย	แทบไม่เคย	บางครั้ง	แทบทุกครั้ง	ทุกครั้ง
30	หนูรู้สึกหงุดหงิดเพราะโรคซีเอ็มทีที่หนูเป็น	☐ 0	☐ 1	☐ 2	☐ 3	☐ 4
31	หนูรู้สึกเหงาเพราะโรคซีเอ็มทีที่หนูเป็น	☐ 0	☐ 1	☐ 2	☐ 3	☐ 4
32	หนูรู้สึกเศร้าเพราะโรคซีเอ็มทีที่หนูเป็น	☐ 0	☐ 1	☐ 2	☐ 3	☐ 4
33	หนูรู้สึกโกรธเพราะโรคซีเอ็มทีที่หนูเป็น	☐ 0	☐ 1	☐ 2	☐ 3	☐ 4
34	หนูวิตกกังวลเพราะโรคซีเอ็มทีที่หนูเป็น	☐ 0	☐ 1	☐ 2	☐ 3	☐ 4
35	หนูไม่มีความสุขกับภาพลักษณ์ของตัวเอง	☐ 0	☐ 1	☐ 2	☐ 3	☐ 4
36	คนอื่นๆ ล้อเลียนเรื่องเท้าหรือมือของหนู	☐ 0	☐ 1	☐ 2	☐ 3	☐ 4
37	หนูกังวลว่าสุขภาพของหนูจะแย่ลงในอนาคต	☐ 0	☐ 1	☐ 2	☐ 3	☐ 4
38	หนูไม่ชอบใจที่จะต้องพึ่งพาความช่วยเหลือจากผู้อื่น	☐ 0	☐ 1	☐ 2	☐ 3	☐ 4
39	หนูมีปัญหาในการจัดการอารมณ์โกรธ	☐ 0	☐ 1	☐ 2	☐ 3	☐ 4

รหัส: ______________________________

<u>โปรดทำเครื่องหมายลงในช่องคำตอบเพียงช่องเดียวของแต่ละข้อความต่อไปนี้</u>

<u>การรู้คิด:</u>

	ระยะหลังมานี้	ไม่เคย	แทบไม่เคย	บางครั้ง	แทบทุกครั้ง	ทุกครั้ง
40	การมีสมาธิเป็นเรื่องยากสำหรับหนู	☐ 0	☐ 1	☐ 2	☐ 3	☐ 4
41	หนูลืมงานที่จำเป็นต้องทำ	☐ 0	☐ 1	☐ 2	☐ 3	☐ 4
42	หนูต้องอ่านบางอย่างหลายครั้งกว่าจะเข้าใจ	☐ 0	☐ 1	☐ 2	☐ 3	☐ 4
43	หนูมีปัญหาในการให้ความตั้งใจ	☐ 0	☐ 1	☐ 2	☐ 3	☐ 4
44	หนูอ่านได้ช้ากว่าเด็กคนอื่น ๆ ที่อายุเท่ากัน	☐ 0	☐ 1	☐ 2	☐ 3	☐ 4
45	หนูมีปัญหาในการหาคำที่เหมาะสมเมื่อพูดคุยกับผู้อื่น	☐ 0	☐ 1	☐ 2	☐ 3	☐ 4
46	หนูมีปัญหาในการติดตามงานของตนเอง	☐ 0	☐ 1	☐ 2	☐ 3	☐ 4
47	คนอื่นเข้าใจหนูได้ยากเมื่อคุยกัน	☐ 0	☐ 1	☐ 2	☐ 3	☐ 4
48	หนูทำงานให้เสร็จทันเวลาได้ช้ากว่าเพื่อน ๆ	☐ 0	☐ 1	☐ 2	☐ 3	☐ 4
49	หนูหงุดหงิดง่ายกับงานอ่านหรือเขียน	☐ 0	☐ 1	☐ 2	☐ 3	☐ 4

รหัส: ______________________________

<u>โปรดทำเครื่องหมายลงในช่องคำตอบเพียงช่องเดียวของแต่ละข้อความต่อไปนี้</u>

***<u>ทักษะทางสังคม</u>*:**

	ระยะหลังมานี้	ไม่เคย	แทบไม่เคย	บางครั้ง	แทบทุกครั้ง	ทุกครั้ง
50	หนูรู้สึกมั่นใจเมื่ออยู่กับเพื่อน	☐ 4	☐ 3	☐ 2	☐ 1	☐ 0
51	หนูรู้สึกมั่นใจเมื่ออยู่กับผู้ใหญ่	☐ 4	☐ 3	☐ 2	☐ 1	☐ 0
52	หนูมีความประพฤติดีเมื่ออยู่นอกบ้าน	☐ 4	☐ 3	☐ 2	☐ 1	☐ 0
53	หนูเข้ากันได้ดีกับคนในครอบครัว	☐ 4	☐ 3	☐ 2	☐ 1	☐ 0
54	หนูมีความสุขเมื่ออยู่บ้าน	☐ 4	☐ 3	☐ 2	☐ 1	☐ 0
55	หนูเข้ากับเพื่อนได้ดี	☐ 4	☐ 3	☐ 2	☐ 1	☐ 0
56	หนูสบายใจที่จะแสดงความคิดเห็น (เกี่ยวกับความรู้สึกของหนูต่อบางสิ่ง)	☐ 4	☐ 3	☐ 2	☐ 1	☐ 0
57	หนูภูมิใจในความมีอิสระ ไม่ต้องพึ่งผู้อื่น	☐ 4	☐ 3	☐ 2	☐ 1	☐ 0
